# A first case association of Lambert-Eaton Myasthenic Syndrome and First Episode Psychosis: a case report

**DOI:** 10.1192/j.eurpsy.2025.2266

**Published:** 2025-08-26

**Authors:** C. Siopa, C. Cordeiro, B. M. Moura

**Affiliations:** 1 Department of Neurosciences and Mental Health, ULS Santa Maria, Lisbon, Portugal; 2 Department of Psychiatry and Neuropsychology, School for Mental Health and Neuroscience, Maastricht University Medical Centre, Maastricht, Netherlands; 3 Católica Medical School, Catholic University of Portugal, Lisbon, Portugal

## Abstract

**Introduction:**

Lambert-Eaton Myasthenic Syndrome is an autoimmune neuromuscular junction disorder characterized by proximal weakness, autonomic dysfunction, and areflexia associated with antibodies against voltage-gated calcium channels. Psychotic symptoms can take place in many auto-immune neurological disorders, but their occurrence in myasthenic syndromes has rarely been observed.

**Objectives:**

We report a case of a 21-year-old female with primary autoimmune Lambert-Eaton Myasthenic Syndrome due to anti-voltage-gated calcium channels antibodies subtype P/Q, who developed psychotic symptoms three years after motor symptom onset.

**Methods:**

The patient attended regular psychiatric follow-ups over three years.

**Results:**

With monthly administration, these psychotic symptoms improved after every cycle of intravenous immunoglobulin therapy. The patient displayed partial insight into the mental symptoms. Different causes of reversible psychosis were excluded, such as autoimmune encephalitis and paraneoplastic syndrome, though the patient tested positive for the anti-voltage-gated calcium channels antibodies subtype P/Q. Owing to muscle strength worsening and psychotic episodes, the patient was put on several treatments, including one admission to a Neurology unit. The patient then experienced psychotic exacerbation, leading to treatment with olanzapine at 20 mg/day. Psychotic symptoms persisted but were less severe, with greater intensity at night. After two years, the patient’s condition showed significant improvement, with olanzapine increased to 25 mg/day.

**Conclusions:**

This is, to our knowledge, the first described case of psychotic symptoms associated with Lambert-Eaton Myasthenic Syndrome. We speculate that voltage-gated calcium channel antibodies could have a role in developing mental symptoms. However, further hypotheses are discussed. Although the patient had received corticosteroid therapy before symptom onset, the timing and dosage make corticosteroid-induced psychosis unlikely. A primary psychotic disorder, such as schizophrenia, is considered improbable due to the atypical nature of the psychotic symptoms. This case underscores the need for further research on the neurobiological mechanisms linking VGCC antibodies to psychiatric symptoms.

**Disclosure of Interest:**

None Declared

